# 356. Impact of CLSI Breakpoint Changes on Unit-Specific Combination Antibiograms for Gram-Negative Respiratory Isolates

**DOI:** 10.1093/ofid/ofad500.427

**Published:** 2023-11-27

**Authors:** Walaiporn Wangchinda, Samuel L Aitken, Virginia M Pierce, Paul Lephart, jason M Pogue

**Affiliations:** University of Michigan College of Pharmacy, Ann Arbor, MI; Michigan Medicine, Ann Arbor, Michigan; University of Michigan Medical School; Michigan Medicine, Ann Arbor, Michigan; University of Michigan, Ann Arbor, Michigan; University of Michigan, College of Pharmacy, Ann Arbor, Michigan

## Abstract

**Background:**

In 2023, the CLSI updated aminoglycoside breakpoints for *P. aeruginosa* and Enterobacterales. The anticipated decrease in aminoglycoside susceptibility rates resulting from these revised breakpoints will impact empiric antibiotic selection for nosocomial pneumonia. This study aims to evaluate the impact of the new breakpoints on the susceptibility rates of various combination antibiotic regimens for Gram-negative pneumonia at Michigan Medicine.

**Methods:**

The susceptibility rates of single and combination antibiotic regimens were determined by applying the CLSI breakpoints to 221 non-duplicated, first Gram-negative respiratory isolates obtained from patients in the MICU and SICU of Michigan Medicine in 2021. Combination antibiograms were developed to compare susceptibility rates of various potential empiric regimens based on the 2022 vs. 2023 aminoglycoside breakpoints. Subgroup analyses for *P. aeruginosa* vs. non-*P. aeruginosa* were performed.

**Results:**

Comparisons of unit-specific combination antibiograms against all Gram-negative respiratory isolates using 2022 vs. 2023 breakpoints are shown in Table 1. Based on the 2022 breakpoints, overall susceptibility rates for aminoglycosides were higher than those of anti-pseudomonal β-lactams. The addition of an aminoglycoside improved the susceptibility percentages of a β-lactam up to 23%, allowing similar activity with amikacin-based combination therapy to levofloxacin-based. In contrast, based on 2023 breakpoints, the susceptibility rates of amikacin and gentamicin dropped significantly (85.1 to 58.8% and 81.0 to 56.6%, respectively) and combination with these two agents did not provide additional benefit to the β-lactam backbone. The tobramycin susceptibility rate was minimally affected by the breakpoint changes (82.8% vs. 79.2%). Subgroup analyses demonstrated that the decrease in susceptibility percentages for aminoglycosides was largely driven by the breakpoint changes for *P. aeruginosa* (Table 1).

**Figure:**
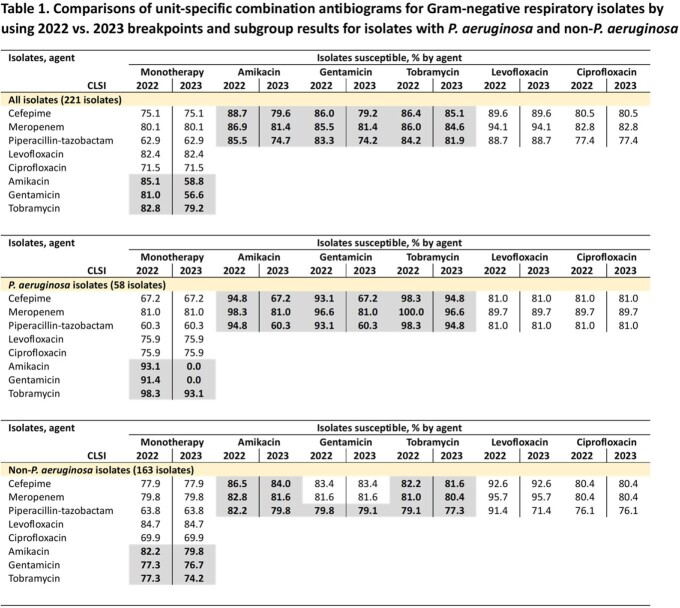

**Conclusion:**

The updated 2023 CLSI breakpoints resulted in a substantial decrease in the susceptibility percentages of amikacin and gentamicin which clinicians need to be aware of when choosing empiric antibiotic treatment.

**Disclosures:**

**Virginia M. Pierce, MD, FIDSA**, UpToDate, Inc.: Authorship royalties **jason M. Pogue, PharmD**, AbbVie: Advisor/Consultant|Entasis: Advisor/Consultant|Ferring: Advisor/Consultant|GSK: Advisor/Consultant|Merck: Advisor/Consultant|Merck: Grant/Research Support|Qpex: Advisor/Consultant|Shionogi: Advisor/Consultant

